# Using wearables to promote physical activity in old age: Feasibility, benefits, and user friendliness

**DOI:** 10.1007/s00391-022-02083-x

**Published:** 2022-07-18

**Authors:** Laura I. Schmidt, Carl-Philipp Jansen, Johanna Depenbusch, Martina Gabrian, Monika Sieverding, Hans-Werner Wahl

**Affiliations:** 1 Institute of Psychology, Heidelberg University, Hauptstr. 47–51, 69117 Heidelberg, Germany; 2 Robert-Bosch-Krankenhaus, Stuttgart, Germany; 3 Deutsches Krebsforschungszentrum (DKFZ), Heidelberg, Germany; 4 Network Aging Research (NAR), Heidelberg University, Heidelberg, Germany

**Keywords:** Behavior change, Self-monitoring, Usability, Acceptance, Health action process approach, Verhaltensänderung, Self-Monitoring, Benutzerfreundlichkeit, Akzeptanz, Health action process approach

## Abstract

**Background:**

Wearables provide new opportunities to promote physical activity also among older adults but data on effectiveness and user friendliness are rare.

**Objective:**

The effects of a comprehensive self-regulative intervention on moderate to vigorous physical activity (MVPA) and number of steps were examined using commercially available activity trackers. Acceptance regarding the devices was analysed in various domains.

**Methods:**

In this study 80 older adults (mean = 67.03 years, standard deviation = 3.97 years; 59% women) wore a Fitbit Charge HR for 21 days including a baseline, a postintervention and a follow-up week. The intervention comprised feedback, goal setting and planning and 50% of the participants were additionally randomized to a role model component. Social cognitive predictors based on the health action process approach (HAPA) and user experience were assessed via questionnaires.

**Results:**

The MVPA increased by an average of 19 min per week and steps by 1317 per day. An additional benefit of the role model component could be observed for MVPA. In the follow-up, the intervention effect was still significant for the number of steps, while MVPA dropped back to baseline. Multilevel models including HAPA variables explained small but significant amounts of variance in MVPA (8% within-person, 26% between-person) and steps (11% within-person, 12% between-person). User experience was rated as very high.

**Conclusion:**

Providing an intervention based on established behavior change techniques and self-monitoring via wearables seems to be effective for increasing physical activity among older adults. The HAPA variables seem to play a limited role to explain activity levels. Acceptance of wearables can be expected to be high.

**Supplementary Information:**

The online version of this article (10.1007/s00391-022-02083-x) contains supplementary material, which is available to authorized users.

## Abstract (de)

**Hintergrund:**

Wearables bieten Potenziale zur Förderung körperlicher Aktivität bei Älteren, jedoch sind Studien, die Effektivität und Nutzerfreundlichkeit gemeinsam betrachten, begrenzt.

**Fragestellung:**

Es werden Effekte einer umfangreichen selbstregulativen Intervention zur Förderung körperlicher Aktivität auf Basis kommerzieller Aktivitätstracker sowie deren Akzeptanz und Bewertung untersucht.

**Methode:**

In dieser Studie trugen 80 Personen (*M* = 67,03; *SD* = 3,97; 59 % Frauen) ein Fitbit Charge HR für insgesamt 21 Tage, einschließlich einer Baseline, einer Post-Interventions- und einer Follow-up-Woche. Die Intervention umfasste Bausteine wie Zielsetzung und Planung, zusätzlich wurden 50 % randomisiert einer Rollenmodell-Komponente zugeordnet. Sozial-kognitive Prädiktoren nach dem Health Action Process Approach (HAPA) und Bewertung der Wearables wurden über Fragebögen erfasst.

**Ergebnisse:**

Moderat bis intensive körperliche Aktivität (MVPA) erhöhte sich nach der Intervention um 19 min/Woche und die Schrittzahl um 1317 pro Tag. Ein Zusatznutzen des Rollenmodell-Ansatzes zeigte sich für MVPA. Im Follow-up blieb der positive Effekt für die Schrittzahl signifikant, während MVPA auf das Baseline-Niveau zurückfiel. Multilevel-Modelle inklusive der HAPA-Variablen („health action process approach“) erklärten kleine aber signifikante Varianzanteile (MVPA: 8 % „within-person“, 26 % „between-person“; Schritte: 11 % „within-person“, 12 % „between-person“). Die Bewertungen (z. B. Nutzerfreundlichkeit) fielen sehr positiv aus.

**Schlussfolgerung:**

Die Intervention auf Basis etablierter Behavior Change Techniken und Self-Monitoring durch Wearables konnte die körperliche Aktivität steigern, wobei Assoziationen zu HAPA-Variablen teilweise bestätigt wurden. Die Akzeptanz und Bewertung der Wearables kann als sehr gut eingestuft werden.

## Introduction

Previous research has established that engagement in regular physical activity is associated with numerous positive effects on physical and mental health among older adults and decreases the risk of functional and cognitive decline [2, 7]; however, only 20–25% of adults aged 65 years and older meet the World Health Organization (WHO) recommendation of exercising at least 150 min per week with moderate or higher intensity [11]. Although the transition to retirement has been recognized as a critical window for promoting physical activity, evidence is inconsistent regarding actually occurring change in patterns [1]. Increasing availability and decreasing costs of activity trackers provide the potential to integrate these devices as a digital enrichment in physical activity interventions. Reviews suggest at least initial positive effects of wearables on physical activity with mostly small to medium effect sizes but are predominantly based on younger cohorts [5, 9, 13]. Older adults have so far received less attention but two recent meta-analyses synthesized respective results of nine [8] and five [16] non-overlapping studies. Overall, wearable trackers were effective in improving physical activity levels among older adults over the short term when compared with usual care or health information [16]. Also, accelerometers, mostly in combination with other co-interventions, increased physical activity, whereas simple pedometers counting steps did not [8].

### Behavior change techniques and the health action process approach

Existing research suggests that only wearing a device does not necessarily increase physical activity. A study based on a 12-month use of an activity tracker did not find changes in total physical activity among recently retired individuals [15]. Instead, it may be necessary to embed the use of wearables within established behavior change techniques [18] to increase physical activity in this key target group for primary prevention efforts.

Hence, our first research aim was to examine the effects of a physical activity intervention based on a selection of three behavior change techniques (feedback, goal setting, and planning) alongside self-monitoring via wearables (Fitbit Charge HR, San Francisco, CA, USA). It is common practice that interventions designed to increase physical activity combine various behavior change techniques, and a systematic review indicated that interventions combining self-monitoring with at least one other technique (e.g., goal setting) were more effective than other interventions [17]. Positive effects of interviewer-assisted action or coping planning were supported by several studies among older adults; however, a review hinted that those with poorer cognitive skills may benefit less from complex behavior change techniques such as planning [12, 21].

As a theoretical framework, we applied the health action process approach (HAPA [19]) to identify social-cognitive determinants of physical activity. The model postulates (1) a motivational phase with risk perception, outcome expectancies, and (action) self-efficacy predicting the intention to engage in a certain health behavior and (2) a volitional phase with intention, (maintenance) self-efficacy, and planning predicting actual behavior. Previous mainly cross-sectional research found some support for the model being able to predict physical activity among older adults; in particular, self-efficacy turned out to be a significant predictor of physical activity intention and behavior in several studies, whereas the evidence for outcome expectancies and risk perception was less consistent [3, 6].

As self-efficacy seems to play a crucial role for physical activity in older adults, the present study additionally evaluated the behavior change technique of vicarious experience through role modelling, a means to increase self-efficacy that implies positive experiences of others who serve as role models. Warner et al. [20] found that vicarious experience was positively related to exercise self-efficacy in older adults and exerted an indirect as well as a direct effect on exercise frequency.

### Acceptance and user experience

Although the integration of wearables in physical activity interventions might come with potential for public health strategies, the bottleneck for successful implementation might be lacking acceptance among older adults. Zhang et al. included participants living in a retirement community (*N* = 40, *M* = 85.4 years, 80% women) who wore a Fitbit Inspire HR for 12 weeks and received goal setting and activity feedback. Findings indicated that participants used the tracker on 97.5% of measured days and that perceived usefulness and ease of use was high [10], in combination with an average increase of 900 steps per day. Brickwood et al. [4] examined user experience regarding the Jawbone UP24 (San Francisco, CA, USA) among older adults with at least one chronic condition across a full year of use (*N* = 20; *M* = 73.6 years, 60% women). Compliance, often used as a proxy for acceptance, was high with the device worn on 86% of possible days. Participants also reported positive experiences in focus groups, i.e., higher motivation related to increased awareness of activity level.

Following these promising but limited findings, our second research aim was to analyze data collected in situ on acceptance and user experience of a commercially available physical activity tracker.

## Method

Ethical approval was obtained by the ethics commission of the Faculty of Behavioral and Cultural Studies at Heidelberg University. Data and analytic code relevant for the reported analyses as well as details on study design and measures are available from the corresponding author upon reasonable request.

### Recruitment and sample

The study was promoted in 2017 via newspaper articles and flyers in the Rhine-Neckar Metropolitan Region as a physical activity intervention study (ActiveAge) for retired adults aged 60+ years who intended to increase their physical activity levels but had not yet realized this goal. A total of 135 individuals were screened via telephone for the following inclusion and exclusion criteria: (1) retired or working less than 10 h per week (including voluntary work), (2) no severe functional limitations, acute pain, or chronic conditions preventing physical activity, (3) no severe visual impairments, (4) no acute depressive episode, (5) no severe cognitive impairment, and (6) no prior experience with activity trackers. Those criteria were met by 85 participants, 5 dropped out before/during baseline due to illness or death of a close family member. Of the final 80 participants, 81% did not meet the WHO guidelines for moderate to vigorous physical activity (MVPA) of at least 150 min per week and 19% reported higher MVPA (*M* = 97.6 min/week, *SD* = 87.3 min/week). Informed consent was obtained from all participants, following detailed information on our strategy to avoid transmission of identifiable data to Fitbit International Limited (see description under “Measures”).

### Procedure and rationale of intervention

The study followed a pre-post intervention without control group design and consisted of a first appointment (T1) followed by the baseline week of physical activity measurement, a second appointment (T2) including the delivery of the intervention followed by the post-intervention week and a third appointment (T3) 3 weeks after T2, again followed by a follow-up week. Alongside self-monitoring that already started at baseline, the following intervention components were delivered by trained staff at T2:

#### Feedback

Participants received individual feedback concerning weekly MVPA and daily steps in the baseline week.

#### Goal setting

Participants were informed about the WHO guidelines for MVPA and the recommendation to walk at least 10,000 steps per day; both served as goals for the remaining study weeks.

#### Planning

Participants were assisted to form action and coping plans to increase physical activity. Action planning included specification of physical activity (what, where, when, and if applicable with whom). Within the scope of coping planning, participants were asked to anticipate potential barriers for the planned activities and how to overcome these.

#### Role modelling

Half of the participants (randomly assigned) watched a 6-min video, in which role models of the same age group from a previous physical activity study talked about their motivation for physical activity and mentioned techniques they had perceived as helpful to overcome barriers. These statements were also handed out in a booklet.

### Measures

Background information assessed at T1 included age, gender, education, body mass index (BMI), subjective health status (excellent = 1 to bad = 5), the functional health subscale of the SF-36 Health Survey (0–100), frequency of smartphone use, and use of health apps (see Table 1). Moreover, general attitudes towards technology were assessed using a 5-item questionnaire [14] (e.g., “If you would like to maintain a modern standard of living, then you must keep pace with technological developments, whether you want to or not”) from 1 (not at all) to 5 (very true).

**Table 1:** Sample characteristics on enrolment

	*M*	*SD*	*N*	*%*
*Age in years*	67.02	3.97	–	–
*Sex*				
Women	–	–	47	58.8
Men	–	–	33	41.2
*Education*				
Primary/secondary school certificate (Grund/Hauptschulabschluss)	–	–	9	11.3
Intermediate secondary school certificate (Realschulabschluss)	–	–	19	23.8
Technical college entrance qualification (Fachhochschulreife)	–	–	15	18.8
University entrance qualification/higher (Abitur/higher)	–	–	34	42.5
Not specified	–	–	3	3.8
*Attitudes towards technology*^*a*^	3.56	0.58	–	–
*Use of smartphone*				
Never/no smartphone	–	–	26	32.5
Several times per week	–	–	5	6.3
Once or twice a day	–	–	24	30.0
Almost every hour/more	–	–	25	31.3
*Use of health apps*				
Never/no health app	–	–	68	85.0
Several times per week	–	–	10	12.5
Once or twice a day	–	–	1	1.3
Almost every hour/more	–	–	1	1.3
*BMI*	27.04	3.75	–	–
*Subjective health*^*b*^	2.92	0.65	–	–
*Functional health*^*c*^	67.71	17.38	–	–

*BMI* body mass index

N = 80

^a^1–5, higher scores indicate more positive attitudes

^b^excellent (1) to bad (5)

^c^0–100, subscale SF-36, higher scores indicate better health

#### Physical activity measurement

At T1, participants were instructed to wear a Fitbit Charge HR (EU certification: Directive 1999/5/EC) during the day and only remove it for water activities. The Fitbit comprises triaxial accelerometry, an altimeter, and heart rate tracking and calculates active minutes in different intensity levels using metabolic equivalents (METs), a measure of energy expenditure, relative to the mass of a person. As raw data are not provided, we used the implemented device algorithm to detect MVPA. Moreover, we used daily step counts as a general indicator for mobility or mainly light physical activity. We created e‑mail aliases and pseudonymous Fitbit accounts in order to avoid transmission of identifying personal data to the company Fitbit International Limited. Moreover, to further ensure data protection and in order not to exclude participants without a smartphone, the Fitbits were not connected to smartphones (i.e., via the Fitbit app). Instead, we transferred activity data directly from the devices to a password-protected computer during the personal appointments. We opted for a controlled setting with only daily steps visible on the tracker.

In addition to Fitbit-measured physical activity, participants filled out daily diaries with respect to the type of physical activity, intensity, duration, and whether they had worn their Fitbit during each activity or not.

#### Health action process approach

We used established scales to assess intention, maintenance of self-efficacy, outcome expectancies and risk perception. Details are given in Supplement 1.

#### Acceptance and user experience

The Telehealthcare Satisfaction Questionnaire for Wearable Technology (TSQ-WT) was used to assess benefit, usability, self-concept, privacy and loss of control, and wearing comfort from 0 (not at all) to 4 (fully agree), for details see Supplement 2. Moreover, we collected information on compliance via the percentage of wearing days and self-reports on non-wearing periods in daily dairies.

### Statistical analyses

Statistical analyses were performed using SPSS 27.0 (IBM, Armonk, NY, USA). All variables displayed a normal distribution, except for MVPA, which was transformed by a logarithm. Multilevel analyses for repeated measures were used to test effects of the intervention alongside the applicability of the HAPA with respect to MVPA and steps. The data structure is hierarchical as observations at measurement weeks (level I) were nested within participants (level II). Pseudo‑R^2^ served as indicator of the effect size.

## Results

The final sample consisted of *N* = 80 participants aged 59–76 years, 63% were married or lived with a partner and all education levels were represented. Attitudes towards technology were relatively positive and 61% used a smartphone at least once a day (see Table 1). Of all sample characteristics, only a higher body mass index (BMI) was associated with lower MVPA (*r* = −0.34, *p* < 0.01) and fewer steps (*r* = −0.32, *p* < 0.01) after the intervention.

Compliance was high in that on average participants wore the Fitbit on 94.8% of the 21 days. After Fitbit data were checked using the daily diaries in terms of plausibility and completeness, 9.8% of study days were considered as invalid days (e.g., forgot to recharge, technical error) resulting in an average of 85.0% valid measurement days.

### Effects of the intervention on physical activity

#### Descriptive results

Although only 19% of participants reported to meet the WHO guideline at enrolment, 47% already reached this level during baseline (MVPA: *M* = 40.6 min/day, *SD* = 48.5; steps: *M* = 10,403.5 steps/day, *SD* = 3336.2). After the intervention, participants increased their physical activity levels (MVPA: *M* = 43.4 min/day, *SD* = 28.5; steps: *M* = 11,720.9 steps/day, *SD* = 3153.7) with 64% meeting the WHO guideline. Although physical activity levels decreased in the follow-up week (MVPA: *M* = 38.8 min/day, *SD* = 35.0; steps: *M* = 11,247.4 steps/day, *SD* = 4175.4), 51% still met the WHO guideline.

#### Testing intervention components and predictors of the health action process approach

Results of the multilevel models are depicted in Table S2 (Supplement 3). For MVPA, a significant effect of the post-intervention week indicated that participants spent more minutes in MVPA after the intervention compared to baseline (*p* < 0.01, *pseudo R*^*2*^ = 0.02); however, this effect vanished during follow-up. A significant effect of the predictor intervention arm indicated that participants who received the role model component performed more MVPA than those who did not (*p* < 0.05, *pseudo R*^*2*^ = 0.03). Adding variables of the HAPA significantly increased the model fit (χ^2^ (10) = 27.82, *p* < 0.01). Positive effects of intention at the interindividual level (*p* < 0.05, *pseudo R*^*2*^ = 0.04) showed that participants with a generally higher intention to be physically active performed more MVPA compared to participants with a lower intention. Overall, the model accounted for 8% of the variance at the within-person level, 26% between-person, and the total variance accounted for was 16%.

The model for steps revealed significant effects of post-intervention and follow-up, indicating that participants walked more steps after the intervention (*p* < 0.001, *pseudo R*^*2*^ = 0.03) as well as 3 weeks later (*p* < 0.05, *pseudo R*^*2*^ = 0.01) compared to baseline. The effect of the role model component did not reach significance. Again, adding variables of the HAPA led to an increased model fit (χ^2^ (10) = 90.00, *p* < 0.001). Furthermore, a negative effect of risk perception at the intraindividual level (*p* < 0.05, *pseudo R*^*2*^ = 0.01) indicated that participants walked fewer steps at measurement weeks with higher risk perception. At the interindividual level, participants with generally higher negative outcome expectancies walked fewer steps (*p* < 0.05, *pseudo R*^*2*^ = 0.04). Overall, the model accounted for 11% of the variance in steps within-person, for 12% between-person and for 15% of the total variance.

### Acceptance and user experience

Descriptive results indicated highly positive evaluations of the TSQ-WT scales benefit (*M* = 3.1, *SD* = 0.81), usability (*M* = 3.5, *SD* = 0.63), self-concept (*M* = 3.4, *SD* = 0.46), and wearing comfort (*M* = 3.2, *SD* = 0.62) alongside very few concerns regarding privacy and loss of control (*M* = 0.4, *SD* = 0.53) regarding the Fitbit. Ratings were not dependent on age, gender, or education. Correlations with change scores in physical activity outcomes indicated a higher increase in steps from baseline to intervention for participants who reported higher benefit (*r* = 0.24, *p* < 0.05), higher usability (*r* = 0.29, *p* < 0.05), and fewer concerns in privacy and loss of control (*r* = 34, *p* < 0.01). For MVPA, higher ratings of usability (*r* = 0.25, *p* < 0.05) and lower privacy issues (*r* = 0.41, *p* < 0.001) were similarly related to a stronger increase, whereas self-concept and wearing comfort were not associated with physical activity outcomes.

## Discussion

The present study investigated the effects of a physical activity intervention based on self-monitoring via Fitbits, feedback, goal setting, and planning among community-dwelling older adults. Findings indicated already increased physical activity levels during baseline, where only the behavior change technique of self-monitoring via wearables was applied, with 47% of participants meeting the WHO guideline according to Fitbit data compared to 19% according to self-reports on inclusion. Despite the restriction of comparing self-reports and Fitbit data, it seems that solely the inclusion plus monitoring via wearables has boosted participants’ motivation to engage in physical activity. On top of these already high physical activity levels, a rather small effect on MVPA after the intervention was found (+18.7 min/week), and a medium-sized effect on steps (+1317/day) compared to baseline. In the follow-up, the effect was only still significant for steps (+844/day). The additional role model component was effective for MVPA, but not for steps. The variables of the HAPA accounted for a significant amount of variance beyond the effect of the intervention, although effect sizes were small. Those small to insignificant associations are in line with Bierbauer et al. [3], who also predicted physical activity longitudinally among older adults and mainly found significant associations in the motivational phase predicting intention in multilevel models, but not for behavior.

Regarding our second aim to explore acceptance and user experience, findings were clearly positive in parallel with low concerns regarding privacy and loss of control. For some dimensions those with more positive experiences exhibited stronger increases in physical activity, although findings cannot be interpreted causally as the TSQ-WT was only assessed once (T3). Moreover, commitment was very high with actual use of the device on 94.8% of measurement days, although some more days had to be coded as invalid after using activity diaries for validation.

Strengths of our study include the combined evaluation of a physical activity intervention and user experience data, a standardized protocol allowing replication, and the combined collection of self-report and Fitbit-based physical activity data; however, several limitations should be considered. First, our sample size was limited and rather homogeneous as participants with more severe functional impairments were excluded. Second, it should be noted that our participants did not need to synchronize the Fitbit with the mobile app, as we aimed to not exclude persons without a smartphone and opted for a controlled setting where the feedback was provided by us. Therefore, TSQ ratings only refer to the device itself and not the Fitbit app. Third, we did not include a control condition without intervention or with self-monitoring only, which is needed to disentangle the effects for efficient intervention design.

Commercially available tracking devices find increasing interest among older adults. Still, evaluative research is needed to explore their potential at large as well as their potential to become a medical product, and by this means gaining the possibility of being reimbursed by health insurances. Therefore, our conclusions are twofold. First, our study contributes to behavior change literature by testing a theory driven intervention targeting physical activity among older adults. Further research should address efficacy in a greater variety of older populations, and longer follow-up periods are needed to determine whether effects can be maintained, and when additional support (e.g., a booster session) is needed. Second, we provide emerging evidence that interventions based on relatively affordable devices may help public health strategies to improve their sustainability.

## Practical conclusion

- Acceptance regarding interventions with activity trackers seems to be high, and user experience is highly positive. Practitioners should encourage older adults with low physical activity levels to take part in respective guided programs.
- As our intervention included several meetings offering rather intense guidance, practitioners should also include individual sessions and be aware of preferences and habits as well as barriers and limitations regarding physical activity.
- We provide one piece of evidence that commercially available trackers may qualify as a promising and cost-efficient medical product („Medizinprodukt“) in near future.

## Supplementary Information

Supplement 1: Assessment of HAPA variables (Health Action Process Approach)

Supplement 2: Sample items for applied subscales of the Telehealthcare Satisfaction Questionnaire for Wearable Technology (TSQ-WT)

Supplement 3: Table S2 multilevel analyses predicting physical activity outcomes

## Declarations

All procedures performed in studies involving human participants or on human tissue were in accordance with the ethical standards of the institutional and/or national research committee and with the 1975 Helsinki declaration and its later amendments or comparable ethical standards. Informed consent was obtained from all individual participants included in the study.
