# Supplementary material for: Using wearables to promote physical activity in old age: Feasibility, benefits, and user friendliness
Source: Z Gerontol Geriatr. 2022 Jul 18;55(5):388–93. doi: 10.1007/s00391-022-02083-x (PMC9360125; doi:10.1007/s00391-022-02083-x)
Supplement: Supplementary file 1 — Supplement 1: Assessment of HAPA variables (Health Action Process Approach) [file 391_2022_2083_MOESM1_ESM.docx]

**Supplement 1: Assessment of HAPA variables (Health Action Process Approach)**

We assessed *intention* using two items adapted from Sieverding et al. (2010), namely, “I intend to be physically active at least 2.5 hours per week” with responses ranging from 0 (not at all) to 6 (completely right), and the subjective probability for realizing this goal (0%– 100%). A mean score with possible scores from 0 to 6 was calculated using the formula (item1+(item2*6/100))/2. For the assessment of *maintenance self-efficacy*, participants were instructed to imagine that they had planned a physical activity. Then, 12 items referring to potential barriers were rated following the intro “I am confident that I can perform the planned physical activity, even if …”, e.g., “I am tired” from 0 (not at all) to 6 (very sure). *Outcome expectancies* were measured with six items for positive and negative expectations, e.g., “If I am physically active for at least 2.5 hours per week”…”I will make new friends” or “I won’t have time for other things” from 1 (not at all true) to 4 (exactly true). *Risk perception* was assessed using a measure of relative vulnerability following Schwarzer (2001). In three items, participants rated their perceived risk regarding chronic pain, mobility limitations, and serious illnesses following the prompt “Compared to an average person of my sex and age my chances of getting ... are” from 1 (much below average) to 5 (much above average).

References:

Schwarzer R (2001) Social-cognitive factors in changing health-related behaviors. Current Directions in Psychological Science 10:47-51. https://doi.org/10.1111/1467-8721.00112

Sieverding M, Matterne U, Ciccarello L (2010) What role do social norms play in the context of men's cancer screening intention and behavior? Application of an extended Theory of Planned Behavior. Health Psychology 29:72-81. https://doi.org/10.1037/a0016941
