# Supplementary material for: Using wearables to promote physical activity in old age: Feasibility, benefits, and user friendliness
Source: Z Gerontol Geriatr. 2022 Jul 18;55(5):388–93. doi: 10.1007/s00391-022-02083-x (PMC9360125; doi:10.1007/s00391-022-02083-x)
Supplement: Supplementary file 2 — Supplement 2: Sample items for applied subscales of the Telehealthcare Satisfaction Questionnaire for Wearable Technology (TSQ-WT) [file 391_2022_2083_MOESM2_ESM.docx]

Supplement 2

*Sample items for applied subscales of the Telehealthcare Satisfaction Questionnaire for Wearable Technology (TSQ-WT)*

TSQ-WT Subscale example items

*Benefit* I can benefit from the Fitbit.

The Fitbit is helping me to achieve my goals.

*Usability* The use of the Fitbit requires effort. (-)

The Fitbit is easy to use.

*Self-concept* The use of the Fitbit is an interesting challenge for me.

I feel embarrassed using the Fitbit visible around others. (-)

*Privacy &* I feel too much supervised by the Fitbit.

*Loss of control* The Fitbit forces me to disclose personal facts that I prefer to keep to

Myself.

*Wearing comfort* Wearing the Fitbit is comfortable.

The Fitbit is difficult to adjust (fix, fast). (-)

*Note:* the TSQ-subscale *quality of life* was not assessed in this study.

All items were rated from 0 (not at all) to 4 (fully agree).

(-) = recoded items

Reference:

Zijlstra W, Clemens B, Klaus P (2011) Wearable systems for monitoring mobility related activities: From technology to application for healthcare services. In: Carsten R, Martina Z (eds) E-Health, Assistive Technologies and Applications for Assisted Living: Challenges and Solutions. IGI Global, Hershey, PA, USA, p 244-267
