# Supplementary material for: Using wearables to promote physical activity in old age: Feasibility, benefits, and user friendliness
Source: Z Gerontol Geriatr. 2022 Jul 18;55(5):388–93. doi: 10.1007/s00391-022-02083-x (PMC9360125; doi:10.1007/s00391-022-02083-x)
Supplement: Supplementary file 3 — Supplement 3: Table S2 multilevel analyses predicting physical activity outcomes [file 391_2022_2083_MOESM3_ESM.docx]

Supplement 3 Table S2

*Multilevel analyses predicting physical activity outcomes*

| Dependent variable | MVPA ^a^ | |  | Steps ^b^ | |
| --- | --- | --- | --- | --- | --- |
| Fixed Effects | b | 95% CI |  | b | 95% CI |
| Intercept | 2.93^***^ | [2.63; 3.25] |  | 9827.68 | [8773.46; 10,881.90] |
| Post-intervention week^c^ | .41^**^ | [ .13; .68] |  | 1302.38^***^ | [594.59; 2010.17] |
| Follow-up week^d^ | .04 | [-.25; .33] |  | 865.09^*^ | [115.19; 1614.99] |
| Intervention arm^e^ | .39^*^ | [-.01; .76] |  | 670.22 | [-417.80; 2249.74] |
| *Intraindividual* |  |  |  |  |  |
| Intention^f^ | -.07 | [-.31; .17] |  | -544.53 | [-1157.30; 68.23] |
| Maintenance self-efficacy^f^ | -.08 | [-.32; .15] |  | -29.45 | [-638.63; 579.73] |
| Pos. outcome expectancies^g^ | -.17 | [-.86; .53] |  | 1149.01 | [-640.98; 2939.01] |
| Neg. outcome expectancies^g^ | -.50 | [-1.10; .10] |  | -823.00 | [-2358.16; 712.16] |
| Risk-perception^h^ | -.03 | [-.34; .28] |  | -821.02^*^ | [-1624.90; -17.15] |
| *Interindividual* |  |  |  |  |  |
| Intention^f^ | .44^*^ | [ .07; .82] |  | 440.90 | [-893.85; 1775.66] |
| Maintenance self-efficacy^f^ | .01 | [-.28; .30] |  | 277.10 | [-738.44; 1292.64] |
| Pos. outcome expectancies^g^ | -.39 | [-.98; .21] |  | 627.15 | [-1473.36; 2727.67] |
| Neg. outcome expectancies^g^ | -.43 | [-1.11; .25] |  | -2532.42^*^ | [-4917.79; -147.06] |
| Risk-perception^h^ | -.01 | [-.26; .29] |  | -27.12 | [-968.60; 932.36] |

*Note.*

For a parsimonious model, random slopes were not included. Variables were centered around the individual mean on Level-I and centered around the grand mean of all participants on Level-II. As all participants received the planning intervention (alongside self-monitoring, feedback, and goal setting) this effect was specified by examining the association between measurement weeks and PA outcomes. Thus, measurement week was dummy coded with the baseline week serving as reference category. To test additional effects of the role-model component, the predictor intervention arm was entered, followed by predictors of the Health Action Process Approach (HAPA). Daily PA measures were averaged for each of the three assessment weeks. Missing information with respect to PA outcomes was not related to sociodemographic or HAPA variables. For both MVPA and steps, reducing the models by eliminating non-significant predictors or adding sociodemographic variables did not improve model fits.

^a^ Fitbit-measured minutes of daily moderate-to-vigouros activity, averaged per measurement week, log transformed

^b^ Fitbit-measured daily steps, averaged per measurement week

^c^ Measurement week 2 =“post-intervention” (dummy coded: 0=baseline, follow-up; 1=post-intervention)

^d^ Measurement week 3 =“follow-up” (dummy coded: 0=baseline, post-intervention; 1=follow-up)

^e^ Intervention arm: 0 = planning only (50% of participants), 1 = planning+role model component (50%)

^f^ from 0 to 6, higher scores indicating higher agreement

^g^ from 1 to 4, higher scores indicating higher agreement

^h^ from 1 (“much below average”) to 5 (“much above average”)

* p < .05, ** p < .01, *** p < .001
